# The impact of green financial development on stock price crash risk from the perspective of information asymmetry in Chinese listed companies

**DOI:** 10.1007/s11356-023-27771-y

**Published:** 2023-07-07

**Authors:** Zhibin Zhang, Youqiang Ding

**Affiliations:** 1 Economic School Shandong Technology and Business University, 191 Binhai Middle Road, Yantai, 264005 Shandong China; 2 School of Finance Tongling University, 1335 Cuihu 4th Road, Tongling, 244061 Anhui China

**Keywords:** Green finance, Stock risk, Information asymmetry, Sustainable development

## Abstract

Solving the crash risk problem of corporate stock price caused by information asymmetry can mitigate the negative externality of its carbon emission to become green, low-carbon, and high-quality development. Green finance generally profoundly impacts micro-corporate economics and macro-financial systems but remains a giant puzzle of whether they can effectively resolve the crash risk. This paper examined the impact of green financial development on the stock price crash risk using the sample data of non-financial listed companies in Shanghai and Shenzhen A stock market in China from 2009 to 2020. We found that green financial development significantly inhibits the stock price crash risk; this is more obvious in listed companies with a high level of asymmetric information. And companies in high-level regions of green financial development attracted more attention from institutional investors and analysts. As a result, they disclosed more information about their operational status, thus reducing the crash risk of corporate stock price from the torrential public pressure of lousy environmental details. Therefore, this study will help continuously discuss the costs, benefits, and value promotion of green finance for synergy between corporate performance and environmental performance to improve ESG capabilities.

## Introduction

The financial policy has not only significantly raised total factor productivity (Chen et al. 2022) but also made investors accustomed to hearing good information and ignoring the crisis possibility, as research by Jin et al. (2019) indicated that economic policy uncertainty affects crash risk through managers’ concealment of negative information and investors’ heterogeneous beliefs. To meet the requirements of shareholders seeking profits, managers usually cover negative information with perfect explanations (Wang et al. 2020). This would produce a stock price crash risk once the negative information was revealed to the public and accelerate the effect of negative externalities from the pollution discharge, i.e., the diesel emission scandal of Volkswagen in 2015 and the environmental data fraud of Bio Agriculture Company 2018. Because the negative information caused extensive media reports in a short period and prompted investors to withdraw their capital, which soon led to the stock price crash risk as the ecological protection hardly by the government. Then ESG (Environmental, Social, and Governance) and CSR (Corporate Social Responsibility) become the strategic choice for enterprises to gain competitive advantages (Zaman et al. 2021). Many kinds of literature proved that corporate performance would be negatively associated with stock price crash risk if managers engage in CSR activities to cover up negative information and divert shareholder scrutiny (Kim et al. 2014). Therefore, green finance has undoubtedly become a primary innovational instrument for managing crash risk in corporate governance (Ding and Hu 2021; Chen and Zhao 2022).

Generally speaking, green finance is vital in promoting the regional economy as a green, low-carbon, and high-quality development road (Wang and Zhou 2022). However, it may generate a crash risk of the corporate stock price should be considered from two aspects: on the one hand, a high-level region of green financial development may attract more institutional investors, analysts, and reports on the environmental performance of enterprises, because the scale of corporate green investment has increased significantly. On the other hand, it will guide more and more institutional investors and analysts to the high-level region of green financial development due to the present policy of “carbon peaking and carbon neutrality goals” in China. Because of many institutional investors and analysts, enterprises dare not act willfully under the law and reduce their hoarding capacity of negative information from the black case. Then it will increase the transparency of external data to effectively reduce the crash risk of corporate stock prices. Previous studies show that enterprises in the high-level regions of green financial development are more willing to disclose more information about their internal activities, which can reflect the business conditions with less asymmetric information (Chen and Zhao 2022). As a result, their stock price crash risk is lower than those of low-level regions of green financial development.

But whether green finance can improve information quality to reduce the stock price crash risk effectively by creating a sustainable financial environment for the real economy remains to be further studied. Following the current research (Wang and Zhou 2022; Xie and Hu 2022), this paper used the sample data of non-financial listed companies in Shanghai and Shenzhen A stock market from 2009 to 2020 to obtain a total score of the green financial development index by entropy method by a negative skewness (NSKEW) and down-to-up volatility (DUVOL) as measurement indicators to empirically study the impact mechanism of green financial development on the stock price crash risk. We found that green financial development can significantly inhibit the probability of the stock price crash risk; the conclusion was also valid after controlling the fixed effects of firms, years, industries, and provinces; it is still consistent after the robustness test when we set up a negative correlation model of green financial development on the stock price crash risk. And the further empirical test of this paper found that institutional investors and analysts tend to pay more attention to companies in high-level regions of green financial development. This conclusion will help limit the information asymmetry between external shareholders and internal companies, reducing the probability of a stock price crash risk.

To prove the above point, we adopt two steps: The first step, we used the *t*-test method to analyze the heterogeneity among institutional investors, analysts, and their reports on listed companies in low-level regions of green financial development compared to those in the high-level areas. The results show more institutional investors, analysts, and their reports on listed companies in the high-level regions of green financial development than those in the low-level areas. In the other step, this paper adopted the *group*-test method to divide two samples to empirically test based on the information asymmetry perspective. We split the High and Low groups by the median value codes according to the composite score for institutional ownership or information transparency; the Yes and No groups by whether the auditor comes from the big four accounting firms. The results show that the negative impact of green financial development on the stock price crash risk is more evident among companies with high information asymmetry. When the information asymmetry is high, the company will benefit more from green financial development, reducing the impact of the information asymmetry between external shareholders and internal companies.

Compared with the existing literature, the possible incremental contributions of this paper are as follows:

First, we aim to expand the literature on green finance by providing additional documentation. Currently, most of the research focuses on measuring the development level of green finance from a single perspective, such as green credit policy (Chen et al. 2023) and green commitment (Liu et al. 2022). To supplement this work, we propose constructing a level of green finance as a new explanatory variable using multiple indicators. This approach will enhance the current research on green finance by providing a more comprehensive view of its development.

Second, we seek to expand the literature on the economic effects of green financial development. While previous studies have primarily focused on the industry and regional levels of green financial development, there has been little exploration of its impact on the micro level. Specifically, this paper investigates the role of green financial development in reducing the crash risk of company stock price, contributing to identifying factors that influence stock price crash risk. Prior research has identified various firm and executive characteristics and other factors that impact stock price crash risk. By studying the level of green financial development, we introduce a new element that may help reduce the crash risk of future stock prices from a fresh perspective. This paper builds upon previous research and adds to the understanding of the economic implications of green finance at the micro level.

Third, previous studies have failed to identify the underlying mechanism linking green finance to corporate stock price crashes. To address this gap, we analyze the impact of green financial development on corporate stock price crash risk from the information asymmetry perspective, allowing for a more comprehensive understanding of its effects. Our findings demonstrate that green financial products can help reduce the crash risk of stock prices and strengthen companies’ commitment to implementing green measures. This paper’s analysis has important implications for environmental protection agencies and individuals, particularly for investors with responsibility for the environment. By shedding light on the mechanism linking green financial development to stock price crash risk, our research will help inform investment decisions considering economic and environmental considerations.

## Literature review

### The definition of stock price crash risk

The crash risk mainly refers to the possibility that corporate stock price will suddenly meet its Waterloo quickly, which will cause its market capitalization to be evaporated instantly (Kim et al. 2014). To make a profit, corporate managers selectively disclose positive information to investors and try to hide adverse information to satisfy the preference of most investors for good news. It is not until the overwhelmed verge of the company that the damaging information will spread quickly like wildfire and immediately appear in front of the panicked investors. That will undermine the investor confidence to impact the stock market’s average regular, making a stock price crash risk come quickly. Therefore, it is essential for the investment decision-making of minority shareholders and the risk decision-making of corporate managers to deeply excavate the stock price risk factors to the attention of investors and regulators (Habib et al. 2018; Dai et al. 2019). In the stock market, portfolio diversification cannot mitigate the crash risk of corporate stock price (Kim et al. 2014); even in business practices, the stock price crash risk is also considered a key determinant of expected returns (Conrad et al. 2013). So the critical economic impact of relevant factors for the stock price crash risk is helpful to a deeper understanding of its determinants for investors and analysts.

From the information asymmetry perspective, there are two hypotheses on the stock price crash risk: the information incompleteness hypothesis and the information hiding hypothesis. Jin and Myers (2006) first proposed the agency theory framework, which suggested that the asymmetric information between managers and shareholders caused a corporate stock price to plummet. Precious studies on the stock price crash risk show that: on the one hand, many factors can increase the probability of the stock price crash risk, such as opaque financial reporting (Hutton et al. 2009; Kim et al. 2019), online platform interactions (Yang et al. 2020), environmental information regulation (Zhang et al. 2022), analyst attention (He et al. 2022), and executive characteristics (Li and Zeng 2019), such as CEO age (Andreou et al. 2017), CEO overconfidence (Kim et al. 2016), and CEO power (Shahab et al. 2020; Al Mamun et al. 2020). On the other hand, in contrast, some factors can help to reduce the probability of the stock price crash risk, such as green investment (Liu et al. 2022), ESG information disclosure (Murata and Hamori 2021), employee stock ownership plan (Li et al. 2019), and oversight mechanisms, such as multiple block-holders (Jiang et al. 2018), industry expert auditors (Robin and Zhang 2015), and analyst coverage (Kim et al. 2019), and so on. However, most scholars still lack research on the critical role of green financial development in stock risk. Therefore, it is worth further studying the impact of green financial development on the crash risk of corporate stock prices while implementing carbon peak and carbon neutrality goals for environmental protection to track more and more extreme climate events in most countries.

### Research progress on stock price crash risk

This study is related to the literature on corporate stock price crash risk. Considering the importance of corporate stock prices, many scholars began investigating factors influencing the stock price crash risk. The factors affecting the stock price crash risk mainly include internal and external factors (Wen 2023). Internal factors include internal governance mechanisms, such as shareholder characteristics (Zhou et al. 2021), director characteristics (Jin et al. 2022), management characteristics (Jiang et al. 2021; Cui et al. 2022), supervisor characteristics (Yin et al. 2022), and other features, as well as internal environmental factors, such as corporate social responsibility (Bae et al. 2021), employee relations (Zuo et al. 2022), and other characteristics. External factors include external governance mechanisms, such as investor protection (Li et al. 2021), media reporting (Zhao 2020), auditor quality (Lim et al. 2016), and other characteristics, as well as external environmental factors, such as oil price uncertainty (Xiao et al. 2022), economic policy uncertainty (Luo and Zhang 2020), political factors (Liu et al. 2023), and other characteristics.

Recently, more and more literature has studied the crash risk of corporate stock prices from an environmental perspective. On the one hand, corporate green behavior will promote corporate stock price rise and reduce the crash risk of stock prices. For example, green technology innovation (Wu and You 2021; Wen 2023), management’s attention to climate change (Jung and Song 2023), disclosure of social responsibility (Ma et al. 2022), the low-carbon transformation of enterprises (Zheng et al. 2022), disclosure of corporate climate risks (Lin and Wu 2023), etc. On the other hand, the impact of changes in the capital market on the stock price crash risk is also crucial. However, most scholars study the green behavior of the capital market from a policy perspective, using quasi-natural experimental methods to analyze the evaluation effect of relevant policies on the stock price crash risk (Zhang et al. 2022; Shao et al. 2022; Chen et al. 2023; Wen 2023). There is relatively little research on the effects of green behavior in the capital market.

### The information asymmetry factors for stock price crash risk

Theoretically, most literature about the stock price crash risk is based on the agency framework in corporate governance. In this framework, the client of investors as an outsider (i.e., the board of directors) relies on control agents (i.e., corporate managers) to access information and to make effective decisions based on the amount and quality of their accessed information (Fama and Jensen 1983). However, the separation of the principals’ ownership and the agents’ control authority raises the question of information asymmetry (Jensen and Meckling 1976). Although the shareholders strongly require that all managers provide timely, fair, and practical management information, managers still do things maliciously for internal corporate strategic or tactical selection reasons. Generally speaking, they tend to report the internal corporate situation by extending the time of information disclosure and trying to reduce the performance problems caused by negative information. So, the information quality obtained by external shareholders is not worth decision-making quickly unless identified carefully. However, managers can not wholly conceal the truth and can only keep this information for a specific period until it must be published (Jin and Myers 2006). Once this false information is inadvertently leaked to the investors, the market will react negatively and cause the corporate stock price to fall sharply.

Since the stock price crash risk is primarily a result of company-specific management characteristics rather than a risk generated by financial assets, clients can not mitigate this risk by diversifying their portfolios (Kim et al. 2014). Therefore, many scholars try to avoid the stock price crash risk by enhancing the quality of corporate information disclosure, considering the factors that do not encourage managers to hoard negative information. Research in this manner suggests that the stock price crash risk may arise from the activities of a company and the nature of its business operations (Habib et al. 2018). For example, (Ben-Nasr and Ghouma 2018) found that more generous employee benefits programs can effectively promote managers’ ability to store information over a more extended period, thus increasing the corporate stock price crash risk. Similarly, Kim et al. (2014) focused on corporate social responsibility commitments and explored whether such obligations would prevent managers from hoarding too much negative information. Their findings contradict the information obfuscation hypothesis, showing more commitment to corporate social responsibility would lead to higher standards of transparency and thus lower the probability of the stock price crash risk. Recently, Wu et al. (2020) research examined the impact of digital finance on the stock price crash risk and found that digital finance significantly reduces the stock price crash risk. This paper further expands these studies and examines the positive impact of green financial development on the stock price crash risk.

### Green financial development

As a new form of financial services, green finance refers to the activities of financial institutions that are required to consider environmental and social factors and to give preferential financing to firms involved in sustainable development (Wen 2023). In 2012, after the Chinese government issued the “Green Credit Guidelines”, many scholars began to explore the evaluation of the Chinese capital market’s green policies on the enterprises’ effectiveness.

On the one hand, under the influence of green finance policy, the debt financing capacity and debt maturity of heavily polluting enterprises have significantly declined (Liu et al. 2023; Wan et al. 2021). In addition, highly polluting enterprises have significantly reduced their capital investment. This has harmed enterprises’ $R \& D$ intensity and total factor productivity (Chen et al. 2001; Wen 2023; Zhang et al. 2022). On the other hand, Zhang et al. (2022), Liu et al. (2023), Shao et al. (2022), and Chen et al. (2023) also studied the impact of stock price crash risk from the perspective of green finance policy. However, most relevant studies choose credit, bonds, or funds to evaluate green finance policies from a single view. As a result, few studies comprehensively consider the impact of green finance development on the stock price crash risk.

## Theoretical analysis and research hypothesis

The profound impact of environmental problems has become increasingly evident (Yao et al. 2021). As a primary pollution source, firms play an essential role in green transformation (Tian et al. 2022). According to signaling theory, regions with high regional green finance development levels provide credible signals of environmental responsibility and financial stability (Spence 1978). Positive signs are deemed reliable and trustworthy. A high level of green financial development in a region indicates that companies are reducing pollutant emissions. The external world can observe the resulting outcomes (such as wastewater, waste gas, etc.), indicating that companies face more stringent external regulations. Under external supervision, corporate management is more motivated to carry out green innovation and production. Therefore, the positive signal of a “high level of green finance development” may make it easier for companies to obtain external financing, leading to less default risk and improved financial returns (Jahmane and Gaies 2020; Lemma et al. 2021), and reputational rewards in terms of corporate brand value and credit ratings (Bruna and Nicoló 2020; Bridge et al. 2020). Existing research indicates that the development of green finance can help enterprises reduce information and agency costs (Rajgopal and Venkatachalam 2011), provide more flexible credit conditions (Liao 2020), and have stronger financial crisis resilience (Wan et al. 2021), thus increasing the stability of corporate stock prices and reducing the risk of stock price crashes.

The stock market has increasingly recognized green financial activities as a new financial instrument in recent years. Green bonds and green credit issued by the company can improve their attention to environmental performance. Thus it attracts more and more institutional investors and analysts to track the green financial company. Considering the company’s contribution to ecological performance, some institutional investors tend to invest in enterprises in high-level regions of green financial development. According to the survey conducted by Eccles and Klimenko (2019) with 70 senior executives from 43 investment institutions worldwide, institutional investors will actively consider whether the target company has done work on environmental performance when choosing the investment direction. Similarly, Park and Jang (2021) pointed out that institutional investors have an investment preference for the target companies marked by ESG governance.

With the formation of the expected value system of green financial development, investors, as the public, pay more and more attention to environmental performance, which affects their information needs in the stock market, and ultimately leads to the preference of analysts for green financial companies (Miroshnychenko et al. 2017; Kim et al. 2019) also found that the probability of the stock price crash risk increased significantly after the decline of analyst attention. Based on the relevant literature, institutional investors and analysts are the main stakeholders influencing corporate strategy and management decisions (McCahery et al. 2016). At the same time, An and Zhang (2013) and Park and Jung (2017) also support this view. They believed system monitoring could ease the manager’s ability to hoard negative information and reduce the probability of a stock price crash risk. Regarding green financial development, Flammer 2020 found that issuing green bonds would effectively improve environmental performance with corporate performance, and the stock market would also respond positively to these green financial companies.

Based on the above analysis, this paper expects enterprises in high levels of green financial development regions will not be prone to the stock price crash risk. And then, we proposed the following assumptions:

### Hypothesis H1

Green financial development is negatively related to the stock price crash risk; green financial development helps reduce corporate stock price crash risk.

## Data sources and description

### The samples

We obtain data from several sources. The green financial development index data comes from the China Statistical Yearbook, Provincial Statistical Yearbook, and the China Insurance Yearbook. Stock and accounting data come from the CSMAR database. The selected samples are processed as follows:

(i) Eliminating the firm sample with abnormal financial status and major anomaly events during the accounting period, such as ST, *ST, etc.
(ii) Eliminating the firm sample with missing values and abnormal values.
(iii) Eliminating the firm sample with less than 30 trading weeks per year to ensure enough days for the crash risk of individual stock prices in the year.

Finally, we got 12008 firm samples in consisting year observations during the 2009-2020 period and winsorized the continuous variables at 1$\%$ above and below them to match the robustness of the results.

### Variable selection

(i) Stock price crash risk

Referring to the related research of Chen et al. (2001) and other scholars, we adopt the negative skewness (*NSKEW*) and the down-to-up volatility (*DUVOL*) based on the extended market model to measure the stock price crash risk. We first regressed the weekly stock returns of each firm sample to the value-weighted market return and used two weekly leading and lagging value-weighted market returns as follows:

1 $$\begin{aligned} r_{i,w}= & {} \beta _{0}+\beta _{1}r_{m,w-2}+\beta _{2}r_{m,w-1} +\beta _{3}r_{m,w}+\beta _{4}r_{m,w+1}\nonumber \\{} & {} +\beta _{5}r_{m,w+2}+\varepsilon _{i,w} \end{aligned}$$

where ${r_{i,w}}$ is the stock return of firm *i* at *w* week, ${r_{m,w}}$ is the value-weighted market index of firm *i* at *w* week, and ${\varepsilon _{i,w}}$ is the error term. To account for asynchronous trading, we include the value-weighted market lag and lead in the estimates (Dimson 1979). Then, we measured firm-specific weekly return for firm *i* at 1 week as the natural logarithm of 1 plus Eq. (1) of the return residual rate:

2 $$\begin{aligned} R_{i,w}=\log (1+\varepsilon _{i,w}) \end{aligned}$$

**Table 1:** Green financial development index

Level I indicator	Level II indicator	Level III indicator
*Green Credit*	Proportion of interest expense in high energy-consuming industries	Interest expenditure of six high energy-energy industries)/(Total industrial interest expenditure)
*Green Investment*	Proportion of investment in environmental pollution control	(Investment in environmental pollution control)/GDP
	Proportion of fiscal expenditure on energy conservation and environmental protection	(Financial expenditure on energy conservation and environmental protection) /(Total financial expenditure)
*Green Bond*	Market value ratio of six high energy-consuming industries	(Total A stock market value of listed companies in high energy-consuming industries) /(Total A stock market value)
*Green Insurance*	Agricultural insurance payout ratio	(Agricultural insurance claims)/ (Agricultural insurance income)
	Agricultural insurance scale proportion	(Agricultural insurance expenditure)/(Total insurance)
*Carbon Finance*	Carbon intensify	(Carbon emissions)/GDP

Based on the skewed situation to capture the asymmetric information of the return distribution, we calculate the negative third moment of the firm-specific weekly return ${R_{i,w}}$ in a single sample year by using the cube of the standard deviation of the firm-specific weekly return as the first proxy variable *NSKEW* for the stock price crash risk.

3 $$\begin{aligned} NSKEW_{s,T}={-}\frac{ \bigg (n(n-1)^{\frac{3}{2}}\sum {R_{i,w}^3} \bigg )}{\bigg ((n-1)(n-2)\big (\sum {R_{i,w}}^{\frac{3}{2}}\big )\bigg )} \end{aligned}$$

Then, we construct the second variable of the stock price crash risk, namely *DUVOL* based on the down-to-up volatility. To avoid the over-influence of extreme weekly returns, we divide the firm-specific weekly returns into two groups: “down weeks” and “up weeks”. The “down weeks” indicate below-average annual returns, and the “up weeks” above average yearly returns. Then, we calculate each sample’s standard deviation of the firm-specific returns. Finally, we take the logarithm of the ratio of the standard deviation of the “down weeks” to the standard deviation of the “up weeks” to capture the value of *DUVOL* as follows:

4 $$\begin{aligned} DUVOL_{i,T}=\log {\frac{\big (n_{up}-1 \big )\sum {_{down}R_{i,w}^2}}{{\big (n_{down}-1 \big )}\sum {_{up}R_{i,w}^2} }} \end{aligned}$$

where ${n_{up}}$ represents the number of “up weeks” and ${n_{down}}$ represents the number of “down weeks” in a fiscal year. To sum up, the higher the value of *NSKEW* and *DUVOL*, the higher the corporate stock price crash risk.

(ii) Green financial development

Referring to the research of Xie and Hu (2022) on the construction method of the green financial development index, we use the entropy method to calculate the level value of green financial development in each province, shown in Table 1.

**Table 2:** Descriptive statistics

Variables	N	Mean	Std	*P*25	*P*50	*P*75	Min	Max
$NSKEW_{t+1}$	12008	$-$0.30	0.71	$-$0.70	$-$0.25	0.13	$-$2.40	1.68
$DUVOL_{t+1}$	12008	$-$0.20	0.48	$-$0.51	$-$0.20	0.12	$-$1.37	1.05
GF	12008	0.25	0.14	0.15	0.22	0.30	0.06	0.79
SIZE	12008	22.05	1.35	21.09	21.86	22.80	17.60	28.51
LEV	12008	0.41	0.20	0.25	0.41	0.57	0.01	0.99
AGE	12008	2.75	0.47	2.48	2.77	3.18	0.69	3.47
OC	12008	36.00	15.49	23.76	34.20	46.70	0.29	89.99
ROA	12008	0.06	2.16	0.04	0.08	0.12	$-$235.10	1.38
PT	12008	0.76	0.43	1.00	1.00	1.00	0.00	1.00
TA	12008	0.92	0.09	0.91	0.95	0.98	0.15	1.00
MTC	12008	94.26	725.50	12.98	26.41	56.04	0.57	38899.00
DI	12008	3.28	1.39	2.31	2.88	3.66	1.15	7.43
FD	12008	0.07	0.04	0.05	0.07	0.08	0.02	0.19

(iii) Control variables

According to the previous research, the control variables in this paper mainly include the corporate and provincial levels.

At the corporate level, the control variables are as follows: $\textcircled {1}$ Firm size (*SIZE*), expressed as the logarithm of the total assets of the listed companies; $\textcircled {2}$ Age of the listed company (*AGE*); $\textcircled {3}$ Liability asset ratio (*LEV*), expressed as the ratio of total liabilities to total assets; $\textcircled {4}$ Ownership concentration (*OC*), expressed by the first shareholder’s shareholding ratio; $\textcircled {5}$ Return on asset ratio (*ROA*); $\textcircled {6}$ Chairman of the board and the CEO (*PT*); $\textcircled {7}$ Tangible assets ratio (*TA*), expressed by tangible assets/total assets; $\textcircled {8}$ Price cash flow ratio (*MTC*), expressed by the percentage of net operating cash flow to market value.

At the provincial level, the control variables are as follows: $\textcircled {9}$ Deposits interest rates of financial institutions (*DI*), expressed by the balance of *RMB* deposits and loans of financial institutions in each province/Provincial GDP; $\textcircled {10}$ Financial development level (*FD*), expressed by the ratio of the GDP of the financial industry in each province to their district GDP. These variable definitions are presented in the Appendix A.

### Descriptive statistics

To get a preliminary understanding of the basic statistical properties of the sample observations corresponding to each variable, Table 2 shows the descriptive statistical results of the relevant variables. The mean values of ${ NSKEW}_{t+1}$ and ${ DUVOL}_{t+1}$ as dependent variables are $-0.30$ and $-0.20$, respectively, and their median values are $-0.25$ and $-0.20$, respectively. These negative values mean the firms have a more left bias in rising weeks than falling weeks. The firm’s specific weekly rate of return and its volatility is slightly higher than that of the falling week, which is consistent with the research of many scholars. The average *GF* of green financial development is 0.25, and their median is 0.30, which is consistent with the research of Wang and Zhou (2022). The descriptive statistics of other control variables were also compatible with the existing literature.

## Empirical results

### Empirical model

To examine the effect of green financial development on the stock price crash risk, we estimate the regression model as follows:

5 $$\begin{aligned} { Stockrisk}_{i,j,t+1}= & {} \alpha _{0}+\alpha _{1}GF_{j,t}+\alpha _{2}Controls_{i,j,t} \nonumber \\{} & {} +\phi _{t}+\mu _{i}+\varepsilon _{i,t} \end{aligned}$$

where *i*, *j*, and *t* refer to firm, province, and year, respectively. The dependent variable is the negative conditional skewness (*NSKEW*) and down-to-up volatility (*DUVOL*). The independent variable is the green financial development index. Control variables consist of Firm size (*SIZE*), Liability asset ratio (*LEV*), Age of the listed company (*AGE*), Ownership concentration (*OC*), Return on assets ratio (*ROA*), and Chairman of the board and the CEO (*PT*), Tangible assets ratio (*TA*), price cash flow ratio (*MTC*), deposit interest rates of financial institutions (*DI*), and financial development level (*FD*). $\phi _t$ is the year fixed effect, $\mu _i$ is the firm fixed effect, and $\varepsilon _{it}$ is the random error term. In all regressions, we report *t*-test with robust standard errors clustered at the corporate level. Considering the possible timeliness problem that the development of green finance affects the crash risk of corporate stock price, this paper deals with the stock price crash risk variable in a lag of one period.

**Table 3:** Stock price crash risk hedging from green financial development

Variables	$NSKEW_{t+1}$			$DUVOL_{t+1}$		
	(1)	(2)	(3)	(4)	(5)	(6)
*GF*	$-$0.556***	$-$0.670***	$-$0.642***	$-$0.262*	$-$0.371**	$-$0.345**
	($-$2.68)	($-$2.62)	($-$2.62)	($-$1.88)	($-$2.08)	($-$2.09)
*SIZE*	$-$0.041***	$-$0.026	$-$0.025	$-$0.039***	$-$0.033**	$-$0.032**
	($-$5.66)	($-$1.16)	($-$1.14)	($-$8.37)	($-$2.09)	($-$2.18)
*LEV*	$-$0.184***	$-$0.288***	$-$0.279***	$-$0.105***	$-$0.149**	$-$0.141**
	($-$4.07)	($-$3.24)	($-$3.31)	($-$3.56)	($-$2.49)	($-$2.48)
*AGE*	$-$0.092***	$-$0.001	$-$0.105	$-$0.066***	$-$0.000	$-$0.099
	($-$3.77)	($-$0.32)	($-$0.90)	($-$4.24)	($-$0.45)	($-$1.26)
*OC*	$-$0.000	$-$0.001	$-$0.001	$-$0.000	$-$0.001	$-$0.001
	($-$0.24)	($-$0.42)	($-$0.60)	($-$0.02)	($-$0.67)	($-$0.92)
*ROA*	$-$0.006	$-$0.009**	$-$0.009	$-$0.008	$-$0.009*	$-$0.008
	($-$0.82)	($-$2.20)	($-$1.02)	($-$1.44)	($-$1.82)	($-$1.40)
*PT*	$-$0.016	0.005	0.005	$-$0.005	0.010	0.010
	($-$0.93)	(0.17)	(0.19)	($-$0.43)	(0.55)	(0.57)
*TA*	$-$0.242***	$-$0.322**	$-$0.325**	$-$0.095*	$-$0.087	$-$0.091
	($-$3.10)	($-$2.49)	($-$2.45)	($-$1.88)	($-$0.95)	($-$1.01)
*MTC*	0.003	$-$0.001	$-$0.002	$-$0.003	$-$0.003	$-$0.003
	(0.05)	($-$0.35)	($-$0.23)	($-$0.80)	($-$0.94)	($-$0.60)
*DI*	$-$0.019	$-$0.018	$-$0.017	$-$0.024	$-$0.026	$-$0.026
	($-$0.68)	($-$0.54)	($-$0.54)	($-$1.28)	($-$1.22)	($-$1.19)
*FD*	0.692	0.922	0.850	0.663	1.000	0.932
	(0.85)	(0.99)	(0.92)	(1.21)	(1.60)	(1.50)
*FirmFE*	NO	YES	YES	NO	YES	YES
*YearFE*	YES	YES	YES	YES	YES	YES
*N*	12008	12008	12008	12008	12008	12008
$R^{2}$	0.0601	0.0612	0.0613	0.0684	0.0692	0.0694

### Baseline regression results

Table 3 reports the empirical analysis results of green financial development on the stock price crash risk. It is found that the levels of green financial development are negatively correlated with stock price crash risk and have statistical significance in all empirical analyses. Significantly, the coefficients of the green financial development (*GF*) in columns (1) and (4) are negative and significant at the 1% level when only the fixed effects of the year, industry, and province are considered. Since firm fixed effects help to explain the constant characteristics over time at the corporate level that may be related to the levels of green financial development on the stock price crash risk, the empirical models that include firm fixed effects are more robust because it alleviates the endogenous problem caused by the bias of neglecting variables. Based on this, the coefficient estimates for green financial development in columns (2) and (5) are also significantly negative at the 5% level when only firm fixed effects are considered. In columns (3) and (6), the empirical model further controls for the fixed effects of the firm, year, industry, and province, and the results are consistent with the previous conclusions.

### The instrumental variables method

In this subsection, we adopt the instrumental variable method (*IV*)and two-stage least squares method (2*SLS*) to test the national low-carbon city pilot policy and the green financial development level in the previous year as instrumental variables to further solve the problem of endogenous and missing variables. A region’s green financial development level usually depends on the country’s policies and procedures. The low-carbon city pilot policies help governments at all levels effectively integrate their green and low-carbon activities into financial services. For provinces and cities that carry out low-carbon pilot projects, more “green” signals will be transmitted and released, and green finance will guide more financial institutions to invest in green financial projects. Therefore, the national low-carbon city pilot policy is more relevant to green financial activities and not directly related to corporate stock price crash risk. We used the national low-carbon city pilot variable ($LC_{dum}$) and the green financial development level with two lags (L2.*GF*) as the instrumental variables of the independent variable *GF*. According to the three batches of national low-carbon city pilot policy documents issued by the National Development and Reform Commission, the national low-carbon city pilot variable ($LC_{dum}$) is dichotomous. If a city is a low-carbon pilot city, the value is assigned to 1; otherwise, it is set to 0.

**Table 4:** Endogenetic test

Variables	$NSKEW_{t+1}$		$DUVOL_{t+1}$	
	$1^{st}$ stage	$2^{nd}$ stage	$1^{st}$ stage	$2^{nd}$ stage
	*GF*	$NSKEW_t+1$	*GF*	$DUVOL_t+1$
$LC_{dum}$	0.005****		0.005****	
	(14.13)		(14.13)	
*L*2.*GF*	0.810****		0.810****	
	(165.99)		(165.99)	
*GF*		$-$1.250****		$-$0.482****
		($-$3.53)		($-$2.03)
*FirmFE*	YES	YES	YES	YES
*YearFE*	YES	YES	YES	YES
*N*	6888	6888	6888	6888
*Ftest*	40539.32****		40539.32****	
$R^{2}$	0.9932	0.0689	0.9932	0.0771
*Sarganstatistic*	0.7647	0.7647	0.4580	0.4580

Table 4 reports the endogeneity test results. In columns (1) and (3), the dependent variables are the first-stage regression results of the levels of green financial development with instrumental variables $LC_{dum}$ and L2.GF as the primary explanatory variable. In the first stage, the coefficient estimates of the low-carbon city pilot variable ($LC_{dum}$) and the level of green financial development (L2.*GF*) with two lags are significantly positive at the 1% level, indicating that the low-carbon city pilot variable ($LC_{dum}$) and the level of green financial development (L2.*GF*) with a lag of two periods are positively correlated with the green financial development. The value of *F*-tests in the first stage is all 40539.32 and is significant at the 1% level, rejecting the hypothesis that the instrumental variable is weak. Therefore, the coefficient estimates in the second stage and their corresponding *t*-statistics may be unbiased. The *P* values of the over-identification test were 0.7647 and 0.4580, respectively, which were insignificant, indicating that the selection of excessive tools was meaningful and met the criteria for instrumental variables.

The results of the second-stage regression are reported in column (2) and column (4) of Table 4. It shows that the estimated coefficient of the green financial development level *GF* is negative and statistically significant at the 5% level in both columns, which is consistent with the benchmark regression results and further supports Hypothesis 1, which means that the green financial development reduces the crash risk of corporate stock price.

### Robustness tests

(i) Digital financial index for a control variable In the green transformation of enterprises, digital finance is crucial for sustainable development (Lang et al. 2021). Firstly, digital finance should be a green development model that reduces energy consumption for enterprises through digital technology, making them more environmentally friendly. Second, digital finance should help improve the service efficiency of green finance, make green finance more accurate, allocate resources more efficiently, and manage risks more effectively. Third, digital finance is conducive to expanding the supply of green finance products and promoting the steady development of the green economy. Industrial structure optimization and green technology innovation by digital finance can have a chain effect on corporate carbon emissions. The geographical spillover effect of digital finance may reduce carbon intensity (Yu et al. 2022). Considering that green financial development may be related to digital finance, we further control digital finance based on the benchmark model. The Digital Financial Index (*DF*) was compiled by a joint research group composed of the Financial Research Center of Peking University and Ant Technology Group from 2011 to 2020. The test results are in columns (1) and (2) of Table 5. The coefficients of the green financial development index (*GF*) are all significantly positive, indicating that the results are still robust after controlling the digital financial index. In addition, compared with the development of digital finance, green financial development has a more significant impact on the risk of a company’s stock price crash.

**Table 5:** Robustness test

Variables	Digital financial variable		Replace DV	Change times	
	$NSKEW_{t+1}$	$DUVOL_{t+1}$	$CRASH_{t+1}$	$NSKEW_{t+1}$	$DUVOL_{t+1}$
*GF*	$-$0.811**	$-$0.416*	$-$0.038***	$-$2.630***	$-$1.407***
	($-$2.46)	($-$1.79)	($-$2.40)	($-$3.01)	($-$2.52)
*DF*	$-$0.002	$-$0.002*			
	($-$1.32)	($-$1.79)			
*FirmFE*	YES	YES	YES	YES	YES
*YearFE*	YES	YES	YES	YES	YES
*N*	10268	10268	12008	5314	5314
$R^{2}$	0.0503	0.0651	0.0367	0.0862	0.119
(ii) Replacement dependent variable We used the dummy variable *CRASH* of stock price crash risk as the dependent variable, which is represented by the indicator function $I{[\bullet ]}$. When the stock *i* of the listed company exists for at least 1 week in a year and satisfies the following inequality, the *CRASH* variable takes a value of 1, indicating that the stock price of the listed company has a crash risk event; otherwise, it is assigned to 0. $\sigma _{i,t}$ is the standard deviation of *i* stock’s holding return in the *t* year, and 3.09 standard deviations correspond to the area where the probability of normal distribution is less than 1%. The empirical results are shown in Table 5, and the conclusion still holds.
  6 $$\begin{aligned} CRASH_{i,T}=I\bullet {\big [ \exists t, W_{i,t} \leqq Average \big (W_{i,t}-3.09\sigma _{i,t} \big ) \big ]} \end{aligned}$$

(iii) Change the time sample In 2015, China proposed “a development concept of innovation, coordination, greenness, openness, and sharing”. Therefore, the data before 2015 of the sample is removed, and the model is re-analyzed empirically. The empirical results are shown in Table 5, and the main conclusions still hold.

## Discussion

### Attention of institutional investors and analysts

This section empirically tested the underlying hypothesis that the attentions of institutional investors and analysts follow the corporate levels of green financial development. When more attention is on these institutions or analysts, limiting a company’s asymmetric information will reduce the probability of the corporate stock price crash risk. To test this hypothesis, we chose a two-step process method: Firstly, we examine whether there are differences in the attention of institutional investors and analysts between high regions and low regions of green financial development; secondly, we examine the formation paths of expected information asymmetry between green financial development and the stock price crash risk. And then, we construct a dummy variable of the green financial development index to examine the average difference in the attention of institutional investors and analysts between high regions and low regions of green financial development ($GF_{dum}$). If the corporate level of green financial development is higher than the median, the indicator variable will take the value of 1; otherwise, it will take the value of 0. We used *t*-tests to obtain the mean differences in institutional investor and analyst attention across the two sample groups (i.e., high regions and low regions of green financial development). Furthermore, we apply a regression model to compare whether companies in the high-level areas of green financial development and low-level parts of green financial development impact the attention of institutional investors and analysts for listed companies.

The *t*-test results reported in Table 6 confirm the significant differences in institutional investor and analyst attention between high-level and low-level regions of green financial development. These results initially support the inference of this paper that institutional investors and analysts tend to focus on companies in high-level regions of green financial development. Institutional investor (*CI*)^1^ and analyst attention (i.e., *analyst* and *RR*)^2^ in the high-level region sample of green financial development is higher than in the low-level region sample of green financial development. The mean difference between the two groups is significant at the 1% level.

To rule out that the difference are not driven by any other corporate-level covariates and to further capture the effect of green financial development on institutional investors’ and analysts’ attention, Table 7 reports the regression results of green financial development on institutional investors’ and analysts’ attention. The results show that the coefficient estimates for the institutional investor (*CI*) and analysts’ attention (*analyst* and *RR*) are positive and statistically significant at the 1% level. These results show that the degree of green financial development positively correlates with institutional investor shareholding and analyst attention to enterprises.

**Table 6:** *T*-test compares regions with high and low green financial developmentt

Variables	$GF_{dum}$=0	$GF_{dum}$=1	*T*-test
*CI*	1.758	2.185	35.652***
*analyst*	7.000	8.000	13.959***
*RR*	11.000	13.000	34.434***
*SIZE*	21.887	21.871	0.868
*LEV*	0.445	0.386	182.937 ***
*AGE*	2.833	2.833	12.436 ***
*OC*	33.340	33.010	1.242
*ROA*	0.071	0.078	40.724***
*PT*	1.000	1.000	0.423
*TA*	0.954	0.954	0.539
*MTC*	26.340	29.143	21.445***
*DI*	2.473	3.576	4738.891***
*FD*	0.054	0.080	5052.656***

**Table 7:** The regression results of green financial development on institutional investors’ and analysts’ attention

Variables	(1) $CI_{t+1}$	(2) $analys_{t+1}$	(3) $RR_{t+1}$
$GF_{dum}$	0.898**	1.493***	3.547***
	(2.50)	(3.15)	(3.16)
*FirmFE*	YES	YES	YES
*YearFE*	YES	YES	YES
*N*	11593	9627	9627
$R^{2}$	0.0835	0.1300	0.0802

### Asymmetric information

The above results report that firms in high-level regions of green financial development receive significantly more tracking from institutional investors and analyst attention than those in the low-level areas. In this context, previous literature suggests that the presence of institutional investors and analysts can reduce asymmetric information by limiting managers’ hoarding of terrible news (McCahery et al. 2016; Zhang et al. 2020). Based on this, we combine empirical analysis with the findings of such literature to show that firms in high-level regions of green financial development will have lower asymmetric information due to timely disclosure of bad news or lower financing constraints due to financing facilitation. We examine the impact paths of the expected asymmetric information between green financial development and stock price crash risk using two steps to provide empirical validity.

Firstly, similar to the process mentioned in the previous section, we used the *t*-test to study the asymmetric information difference between high-level and low-level firms of green financial development. The *t*-test results are in Tables 8, 9 and 10. The results show significant differences in the variables of asymmetric information between the two groups. That is, there are substantial differences in asymmetric information between high-level and low-level firms of green financial development. These results initially support that high-level firms of green financial development have asymmetric lower information than low-level firms.

Secondly, drawing on the research of Xin et al. (2014), we used transparency indicators to measure asymmetric information for enterprises, investors, and analysts. We divide the transparency indicators into three variables: institutional investor shareholding, whether the auditor is from the big four accounting firms, and the total score transparency score.^3^

Furthermore, the method of group test was used to divide the whole sample into high firms of information asymmetry (the shareholding ratio of institutional investors and the total transparency score are higher than its median value, and the auditors are from the big four accounting firms). For companies with low information asymmetry (the shareholding ratio of institutional investors and the composite transparency score are lower than its median value, and the auditors are not from the big four accounting firms), the benchmark regression model is re-run on the two subsamples, respectively.

Table 8 reports the results of green financial development and stock price crash risk for institutional investor holdings’ lower and higher subsamples. The results show that, in columns (1) and (3), green financial development negatively correlates with the corporate stock price crash risk with a lower shareholding ratio by institutional investors. In contrast, in columns (2) and (4), the coefficient of green financial development is insignificant. This means that green finance in companies with a low ratio of institutional investors can effectively curb the stock price crash risk. In contrast, institutional investors themselves have a substantial supervisory role. Therefore, firms with higher shareholding by institutional investors are more transparent (Sun and Yang 2017), so firms with higher shareholding by institutional investors are green financial companies. The role of development is not apparent.

**Table 8:** *Group* test for institutional investors

Variables	$NSKEW_{t+1}$		$DUVOL_{t+1}$	
	(1) *Lowgroup*	(2) *Highgroup*	(3) *Lowgroup*	(4) *Highgroup*
$GF_{dum}$	$-$0.798*	$-$0.448	$-$0.538*	$-$0.180
	($-$1.73)	($-$1.20)	($-$1.72)	($-$0.68)
*FirmFE*	YES	YES	YES	YES
*YearFE*	YES	YES	YES	YES
*N*	5357	6651	5357	6651
*Ftest*	40539.32***		40539.32***	
$R^{2}$	0.0613	0.0742	0.0695	0.0811
$t-test$	116.156***	116.156***	75.765***	75.765***

**Table 9:** *Group* test for audits from big four accounting firms

Variables	$NSKEW_{t+1}$		$DUVOL_{t+1}$	
	(1) *NOgroup*	(2) *YESgroup*	(3) *NOgroup*	(4) *YESgroup*
$GF_{dum}$	$-$0.769***	$-$0.924***	$-$0.465***	$-$0.285***
	($-$2.66)	($-$1.36)	($-$2.30)	($-$0.60)
*FirmFE*	YES	YES	YES	YES
*YearFE*	YES	YES	YES	YES
*N*	11102	906	11102	906
$R^{2}$	0.0610	0.1300	0.0695	0.1520
$t-test$	37.783***	37.783***	37.090***	37.090***

**Table 10:** *Group* test for transparent comprehensive index

Variables	$NSKEW_{t+1}$		$DUVOL_{t+1}$	
	(1) *Lowgroup*	(2) *Highgroup*	(3) *Lowgroup*	(4) *Highgroup*
$GF_{dum}$	$-$1.119**	$-$0.190	$-$0.661*	$-$0.010
	($-$2.13)	($-$0.54)	($-$1.87)	($-$0.04)
*FirmFE*	YES	YES	YES	YES
*YearFE*	YES	YES	YES	YES
*N*	5497	6401	5497	6401
$R^{2}$	0.0600	0.0731	0.0737	0.0771
$t-test$	22.368***	22.368***	8.827***	8.827***

Table 9 shows the impact of green financial development on whether auditors from the big four accounting firms group on the share price crash risk. In columns (1) and (3), when the auditors are from general accounting firms other than the big four accounting firms, the impact of green financial development on stock price crash risk is significantly negatively correlated. In contrast, in columns (2) and (4), the coefficient of green financial development in companies whose auditors are from the big four accounting firms is insignificant. It shows that green financial development can significantly suppress the corporate stock price crash risk whose auditors are from general accounting firms. In contrast, the quality of financial reports issued by the big four accounting firms is relatively high for those whose auditors come from the big four accounting firms. The company chooses the big four accounting firms as the audit department, which signals that it is willing to promise to provide truthful financial information (Lang and Maffett 2011), and the role of green financial development is not prominent.

Finally, Table 10 reports the relationship of transparency to green financial development and stock price crash risk. In columns (1) and (3), the impact of green financial development on stock price crash risk is significantly negatively correlated in the less transparent groups; in columns (2) and (4), in the more transparent groups, The role of green financial development in the group is not apparent. To sum up, the test results in Table 10 are consistent with the theoretical analysis of this paper. That is, developing green finance helps promote corporate information transparency and reduce the probability of stock price crash risk.

Overall, the test results reported in Tables 5, 6, 7, 8, 9, and 10 confirm that institutional investors and analysts tend to pay more attention to companies in areas with higher levels of green financial development. The presence of institutional investors and analysts can reduce the internal and external relationship between listed companies. The information asymmetry between them can help reduce the stock price crash risk.

## Conclusions

We used the stock price data of listed companies in Shanghai and Shenzhen A stock markets and the green financial development data in various provinces of China from 2009 to 2020 to examine the impact of green financial development on the stock price crash risk. This study found that green financial development negatively correlates with the stock price crash risk. The research results are consistent after a series of robustness tests. Furthermore, green financial development attracts the attention of institutional investors and analysts by exploring the potential paths for green financial development to affect the corporate stock price crash risk. This can reduce managers’ hoarding capacity of bad news to avoid the stock price crash risk. In particular, institutional investors and analysts prefer firms in high-level regions of green financial development significantly more than those in low-level areas. Compared with low-level firms of green financial development, those high-level firms have lower information asymmetry. In addition, green financial development has a more significant inhibitory effect on firms with a higher degree of information asymmetry, especially in the insufficient corporate transparency of lower shareholding by institutional investors and auditors from accounting firms to hedge the stock price crash risk. Therefore, a key path for green financial development to reduce the stock price crash risk is the reduction of corporate information asymmetry.

The research conclusion has important policy implications for promoting the construction of a green financial system and promoting the high-quality development of enterprises: (i) Green financial development in China is in its infancy. For this effect, the government should vigorously strengthen policy support for green finance, improve the corporate efficiency of green financial funds, and build a long-term mechanism for national green financial development. (ii) Give full play to the professional roles of institutional investors and analysts in green finance instruments to form an incentive mechanism for the green financial development of enterprises. (iii) Relevant departments should improve the laws and regulations on green finance and low-carbon development of enterprises to ensure that institutional investors and analysts can participate in the long-term and regular supervision of their operation and management.

## Data Availability

The data can be available on request

## Appendix A: Variables definition

The three variables in this model are explained as follows:

(i) Dependent variable: **Stock price crash risk**. *NSKEW *: Negative conditional skewness is a ratio of weekly returns of the third moment in each firm year to the cubic standard deviation of weekly returns and multiplies that ratio by $-1$; see Eq. (3). *DUVOL*: Down-to-up volatility is the natural logarithm of the ratio of the standard deviation in the “down” weeks to the standard deviation in the “up” weeks; see Eq. (4).
(ii) Independent variable: **Green financial development**. *GF*: The legal weight of the entropy method was used to obtain the total score of the green financial development index according to the construction method of Xie and Hu (2022).
(iii) Control variables

### Firm size

*SIZE*: Natural logarithm of the book value of total assets(in RMB) at the end of the year.

### Liability asset ratio

*LEV*: The percentage of total liabilities to total assets.

### Age of the listed company

*AGE*: The listed company’s establishment years logarithmic.

### Ownership concentration

*OC*: The book value of total debts divided by the book value of total assets.

### Return on assets ratio

*ROA*: Ratio of net cash flows from operating activities to total assets.

### Chairman of the board and the CEO

*PT*: A dummy variable that equals one if the CEO is also the chairman of the board and 0 otherwise.

### Tangible assets ratio

*TA*: The balance of the total tangible assets to the enterprise’s total assets.

### Price cash flow ratio

*MTC*: The net operating cash flow ratio to a listed company’s market value.

### Deposit interest rates of financial institutions

*DI*: The ratio of RMB deposits and loans of financial institutions in the province to the region’s GDP.

### Financial development level

*FD*: The ratio of the financial GDP of each province to the region’s GDP.

## Footnotes

1 The variable of institutional investor is mainly measured by the shareholding ratio of institutional investors in listed companies, and the data comes from the *CSMAR* database.

2 The variable of analyst attention is mainly measured by the analysts’ track numbers and research report monitoring analyses, and the data comes from the *CSMAR* database.

3 Referring to the practical method of Xin et al. (2014), we used the average sample percentage grades of five variables, including earnings quality, information disclosure score, analysts track numbers, analysts’ earnings forecast deviation, and auditors from the big four accounting firms. The value of the calculated result is the composite transparency score. The higher the composite transparency score, the higher the company’s information transparency.
